# Closure of oroantral communication with buccal fat pad after removing bilateral failed zygomatic implants: A case report and 6-month follow-up

**DOI:** 10.4317/jced.51741

**Published:** 2015-02-01

**Authors:** David Peñarrocha-Oltra, Rocio Alonso-González, Hilario Pellicer-Chover, Amparo Aloy-Prósper, María Peñarrocha-Diago

**Affiliations:** 1 Master in Oral Surgery and Implant Dentistry. Collaborating Professor of Oral Surgery, Stomatology Department. Faculty of Medicine and Dentistry. University of Valencia, Spain; 2 Full Professor of Oral Surgery. Stomatology Department. Faculty of Medicine and Dentistry. University of Valencia, Spain

## Abstract

The aim of this study was to assess the use of buccal fat pad (BFP) technique as an option to close oroantral communications (OAC) after removing failed zygomatic implants in a patient with a severely resorbed maxilla, and to determine the degree of patient satisfaction. A 64-year-old woman presented recurrent sinusitis and permanent oroantral communication caused by bilateral failed zygomatic implants, 3 years after prosthetic loading. Zygomatic implants were removed previous antibiotic treatment and the BFP flap technique was used to treat the OAC and maxillary defect. The degree of patient satisfaction after treatment was assessed through a visual analogue scale (VAS). At 6-months follow-up, patient showed complete healing and good function and the results in terms of phonetics, aesthetics and chewing were highly rated by the patient.

** Key words:**Bichat fat pad, buccal fat pad, zygomatic implants, oroantral communication.

## Introduction

Several techniques have been described to treat the atrophic maxilla (Cawood and Howell classes IV or V) (1), including zygomatic implants (ZIs) (2). Although ZIs seemed to have high survival rates, complications are common (2), as permanent oroantral fistula formation (3) that may be responsible for recurrent sinusitis and therefore, indication for ZI removal (3). Numerous techniques for oroantral communication (OAC) closure, including grafts and flaps of proximity or distance, such as pedicled Bichat´s ball (BFP) have been described (4).

Since in 1977 Egyedi (5) described the technique of closure oroantral fistula by using pedicled Bichat´s ball, it has become a procedure widely used in regenerative oral surgery. In the past four decades, several authors have resorted to using the Bichat´s ball to close oroantral communications of diverse etiology (5-9) either acute, chronic or recurring character (9). The reported advantages of its use have been the easy availability of the flap, and the large blood supply that the recipient bed receives, resulting in high success rates (6,10). Complications of this technique are rare (4,11), resulting in most cases aesthetic, phonetic and chewing acceptable results.

A clinical case is reported in which the BFP technique was used to OAC closure after removing failed zygomatic implants in a patient with a severely resorbed maxilla, and to determine the degree of patient satisfaction.

## Case Report

A 64-year-old woman referred discomfort in the maxillary area. Clinical history examination revealed that the atrophic maxilla was rehabilited four years before, by 3 conventional implants implants (Phibo® TSA, Phibo Dental Solutions, Impladent, Senmenat, Barcelona, España), two zygomatic implants (Nobel Biocare®, Goteborg, Sweden) and fixed full-arch implant-supported prosthesis. After three years of loading, bilateral sinusitis have been diagnosed (the patient had rhinorrhea, cacosmia, and pain in malar area) and treated through Cadwell-Luc technique and antibiotic treatment (Proflox 400mg, 1 compressed, once daily during 7 days).

**Figure 1:**
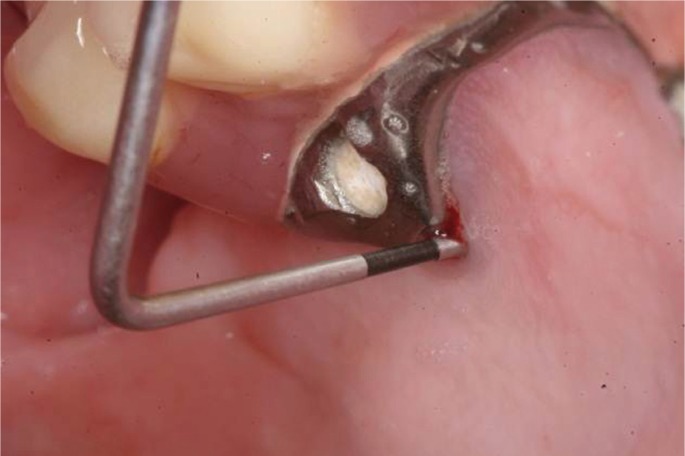
Oroantral communication in relation to zygomatic implants (ZIs) in a severely resorbed maxilla, after 3 years of prosthetic loading. Detail of probing depth verifying the permanent bilateral OAC before ZIs extraction.

**Figure 2:**
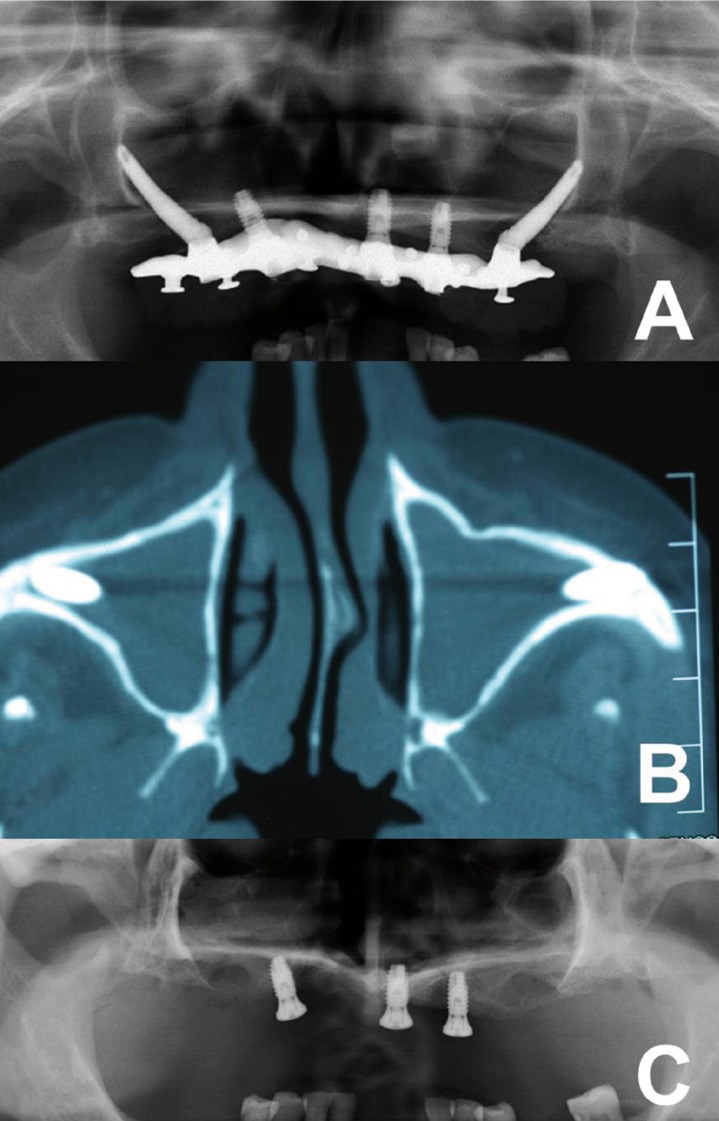
A)Panoramic radiography and B) computerized tomography (TC) showing bilateral maxillary sinus occupation (sinusitis) secondary to permanent oroantral communication due to resorption of the thin palatal bone corresponding to ZIs. C) Control orthopantomography 6 months after surgery.

One year after sinusitis treatment, recurrent sinusitis was diagnosed. During clinical examination and questionnaire, the patient reported inaccurate pain at bilateral sinus level of varying intensity. Patient had slight tenderness of cheekbones. Colour and texture of the gum was correct, but at probing depth, the zygomatic implants showed palatal oroantral communication (Fig.1). A radiographic examination (panoramic radiography and computerized tomography [CT], Fig. 2a-b) was performed. Oroantral communication accompanied by bilateral recurrent sinusitis was diagnosed.

Amoxicillin 500 mg/125 clavulanic acid, 3 times during 10 days, and Ibuprofen (600 mg, 3 times daily) were prescribed for the treatment of sinusitis and pain. Patient was revaluated one month later. Due to OAC permanence and recurrent sinusitis history, removal of both ZIs was decided. OAC closure through buccal fat pad flat technique was planned as it is described in the literature.

-BFP technique

**Figure 3:**
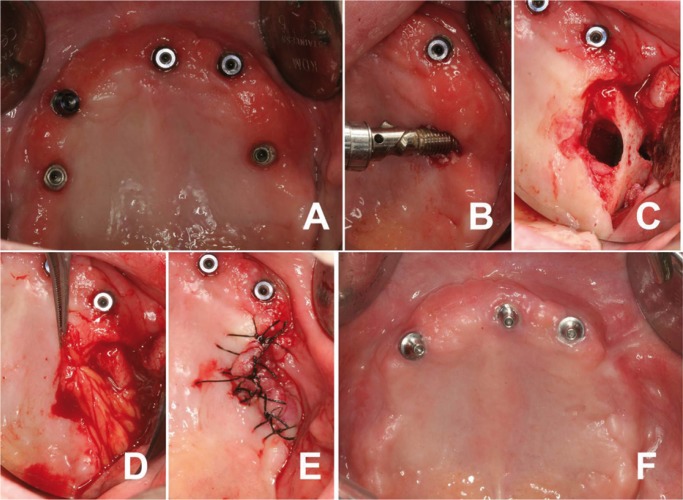
Surgical treatment. Detail of left ZIs removal and surgical OAC closure through buccal fat pad flap technique. A) Intraoral clinical picture prior to failed ZIs extraction. B) Left ZI removal. C) Mucoperiosteal flap elevation showing orosinusal communication. D) Pedicled buccal fat covering the maxillary defect area. E) Mucoperiosteal flap replacement and suture. F) Maxillary gingiva healing 6 months after surgery.

Once recurrent sinusitis was resolved, ZIs were removed (Fig. 3a-e). Operation was performed by an experienced surgeon (MP). After local anesthesia with articaine and infiltrative 4% and adrenaline 1:100.000 (Inibsa ®, Lliça Vall, Barcelona, Spain), ZIs were removed and a trapezoidal mucoperiosteal flap was obtained by two divergent incisions, one on each side of the location of the defect, extending to the bottom of the vestibule. The COA defect was exposed (Fig. 3c). BFP was harvested by performing a 1-cm crestal incision starting at the tuberosity behind the zygomatic buttress. Then, a blunt clamp was introduced to the temporomandibular angle in order to separate the fibers of the buccinator muscle. By a slight pressure on the cheek, the buccal extension of Bichat´s ball was exposed. The necessary amount of buccal fat was pedicled to entirely cover the defect area (Fig. 3d). BFP was covered as much as possible by the mucoperiosteal flap and it was sutured without tension (Fig. 3e). Analgesics and antibiotic prophylaxis was prescribed (Amoxicillin 500mg + clavulanic acid 125mg every 8 hours for 7 days). A soft diet was recommended for 1 week and the patient was instructed to avoid brushing and trauma on the surgical sites. Sutures were removed 1 week postoperatively. Conventional denture was confectioned and was worn provisionally in the healing periods.

-Follow-up and patient satisfaction 

The patient was screened in a program of routine check-ups (one week, 1 and 6 months after surgery). No postoperative complications were collected on successive controls. An overdenture over 3 residuals implants was performed as a new prosthesis design. At six months of follow-up after surgery, patient showed complete healing and the oroantral communication had been resolved (Fig. 2c-3f).

At 6-months follow-up, patient satisfaction was assessed in order to determine overall satisfaction regarding treatment and new prosthesis design. A ten-cm visual analogue scale (VAS) (range 1-10) was used to estimate patient satisfaction. General satisfaction with the implant-retained prosthesis and specific satisfaction regarding aesthetics, phonetics and mastication were assessed. The patient was asked to draw a vertical line at a point on the horizontal line which best represented his response (12,13). The best valued parameter by patient was the phonetic [9], followed by chewing [8] and aesthetics [7]; the mean overall satisfaction was 8 out of 10.

## Discussion

Literature provides high ZIs survival rates; however, this type of implants is not free of complications (2,14). Some authors have reported ZIs removals because of recurrent sinusitis which were not resolved with antibiotics and sinus rinses (3). In some cases, this sinus infection is secondary to oroantral fistulae formation, which is speculated to appear due to deficient osseointegration of the coronal part of the ZI, thereby creating the communication between the oral and sinus cavities (3,14).

Resorption of the thin palatal bone rapidly leads to oroantral fistula followed by implant loss (3), and it seems likely to occur at any time after implant placement (2). In the present case report, ZIs extraction was decided due to recurrent sinusitis history and persistent oroantral communication (OAC) 3 years after prosthesis loading.

One important question in the case reported was the OAC management. Bilateral buccal fat pad (BFP) flap technique to solve the maxillary defect was decided.

The BFP is an adipose mass located in the deep facial spaces. It has been widely used to reconstruct oral and maxillofacial defects because of its physical and biological properties, e.g.: its anatomical location closest to the recipient bed, vascularization, ease of production and management, and the presence of stem cells (9,10,15). Some researchers have recommended the BFP flap as a first option for closure of larger OACs (4,6,7).

The most critical factor for the success of the buccal fat pad seems to be the communication´s size (10); Abuabara *et al.* (4) re-commended the use of the Bichat´s ball in large communications (> 5 mm in diameter), in which the use of buccal flap could compromise its blood supply and/or loss of vestibular sulcus depth. However, limiting the amount of pedicled Bichat´s ball is recommended because large defects require greater traction of the pedicle, and it may increase postoperative complications such as aesthetic depression of the cheek (6). Most common complications in the literature were the persistence of the fistula and limitation of mouth opening, especially after reconstructing oroantral communications accompanied by large bone defects (6,8). However, most studies have shown good results with BFP´s technique to close oroantral communications and treat maxillary bone defects (4,6-9,11). The advantages of BFP graft include the easy access to the anatomic region for excision, and the large blood supply that the recipient bed receives, yielding high success rates in OAC closure (6,10). To our knowledge, this is the first BFP´s case reporting patient satisfaction and assessing the changes after surgery regarding to aesthetics, phonetics and mastication.

In this case report, the use of BFP was a good treatment option to close oroantral communications caused after removing failed zygomatic dental implants and neither recurrences nor complications were found. At six months of follow-up after surgery, patient showed complete healing and good function. The results in terms of phonetics, aesthetics and chewing were highly rated by the patient.

## References

[1] Cawood JI, Howell RA (1988). A classification of the edentulous jaws. Int J Oral Maxillofac Surg.

[2] Chrcanovic BR, Abreu MH (2013). Survival and complications of zygomatic implants: a systematic review. Oral Maxillofac Surg.

[3] Becktor JP, Isaksson S, Abrahamsson P, Sennerby L (2005). Evaluation of 31 zygomatic implants and 74 regular dental implants used in 16 patients for prosthetic reconstruction of the atrophic maxilla with cross-arch fixed bridges. Clin Implant Dent Relat Res.

[4] Abuabara A, Cortez AL, Passeri LA, de Moraes M, Moreira RW (2006). Evaluation of different treatments for oroantral/oronasal communications: experience of 112 cases. Int J Oral Maxillofac Surg.

[5] Egyedi P (1977). Utilization of the buccal fat pad for closure of oro-antral and/or oro-nasal communications. J Maxillofac Surg.

[6] Poeschl PW, Baumann A, Russmueller G, Poeschl E, Klug C, Ewers R (2009). Closure of oroantral communications with Bichat's buccal fat pad. J Oral Maxillofac Surg.

[7] de Moraes EJ (2008). Closure of oroantral communication with buccal fat pad flap in zygomatic implant surgery: a case report. Int J Oral Maxillofac Implants.

[8] Abad-Gallegos M, Figueiredo R, Rodríguez-Baeza A, Gay-Escoda C (2011). Use of Bichat's buccal fat pad for the sealing of orosinusal communications. A presentation of 8 cases. Med Oral Patol Oral Cir Bucal.

[9] Dolanmaz D, Tuz H, Bayraktar S, Metin M, Erdem E, Baykul T (2004). Use of pedicled buccal fat pad in the closure of oroantral communication: analysis of 75 cases. Quintessence Int.

[10] Singh J, Prasad K, Lalitha RM, Ranganath K (2010). Buccal pad of fat and its applications in oral and maxillofacial surgery: a review of published literature (February) 2004 to (July) 2009. Oral Surg Oral Med Oral Pathol Oral Radiol Endod.

[11] Hernando J, Gallego L, Junquera L, Villarreal P (2010). Oroantral communications. A retrospective analysis. Med Oral Patol Oral Cir Bucal.

[12] Heydecke G, Boudrias P, Awad MA, De Albuquerque RF, Lund JP, Feine JS (2003). Within-subject comparisons of maxillary fixed and removable implant prostheses: Patient satisfaction and choice of prosthesis. Clin Oral Implants Res.

[13] Pjetursson BE, Karoussis I, Bürgin W, Brägger U, Lang NP (2005). Patients' satisfaction following implant therapy. A 10-year prospective cohort study. Clin Oral Implants Res.

[14] Aparicio C, Ouazzani W, Garcia R, Arevalo X, Muela R, Fortes V (2006). A prospective clinical study on titanium implants in the zygomatic arch for prosthetic rehabilitation of the atrophic edentulous maxilla with a follow-up of 6 months to 5 years. Clin Implant Dent Relat Res.

[15] Farré-Guasch E, Martí-Pagè C, Hernádez-Alfaro F, Klein-Nulend J, Casals N (2010). Buccal fat pad, an oral access source of human adipose stem cells with potential for osteochondral tissue engineering: an in vitro study. Tissue Eng Part C Methods.

